# Early detection of hypoxia in COVID-19

**DOI:** 10.11604/pamj.supp.2020.35.2.23871

**Published:** 2020-06-04

**Authors:** Sa’ad Lahri

**Affiliations:** 1 Anesthesiology and ICU Department, University Hospital of Casablanca, Morocco

**Keywords:** COVID-19, hypoxia, early detection

## To the Editors of the Pan African Medical Journal

Patients with coronavirus disease 2019 (COVID-19) may present with a clinical picture of severe acute respiratory illness. Acute respiratory failure may complicate COVID-19 pneumonitis, of which the dominant feature may be severe hypoxaemia [1]. The COVID-19 pandemic will lead to a surge in the number of critically ill patients who may require additional ventilator support [2]. Resources in low- and middle-income countries are limited and early screening, detection, diagnosis and treatment will mitigate morbidity and mortality. Intubation and mechanical ventilation may have limited impact on outcome, with mortality as high as 88% in one large cohort of patients in New York [3]. Severe covid-19 leads to a prolonged hospital stay and a high mortality rate [4]. Hypoxia early in hospital admission was an important marker for adverse events [5].

In order to reduce the impact of COVID-19 severity of presentation and reprioritise the focus on improving patient outcomes a proposal is made for a process whereby patients and their families are taught the signs of increased work of breathing, how to count their own respiratory rate and the use of pulse oximetry for patients with a known diagnosis of SARS-CoV-2. COVID-19 has a specific clinical challenge: patients who are hypoxic but do not display overt signs of respiratory distress or increased breathing efforts, often referred to as the “silent” or “happy” hypoxic patient. The principal characteristic is the dissociation between the severity of the hypoxaemia and the maintenance of relatively good respiratory mechanics [1]. Healthcare workers may ask patients who are suitable for discharge or those whose results are positive to monitor their respiratory rates (trigger of 20 breaths per minute). Patients and their families may be taught to identify the phasic contraction of the sternocleidomastoid muscle, which is the most direct sign of increased breathing effort, during a physical examination [1].

Also, the patient may be given a pulse oximeter, and these readings may be sent through to the clinical and or monitoring team.Patients will thus be able to screen their saturation levels as soon as they fall to below 95%. They then can activate the emergency medical services and thus prevent complications associated with delayed presentations. Pulse oximetry in this setting may identify patients who are deteriorating sooner and thus prompt them to seek earlier medical intervention. This may not be completely feasible due to resource constraints so another option could be community workers doing the screening when patients are being monitored at home. Patients would also be need to be trained in the correct use and cleaning of a pulse oximeter. A caveat to this is that some patients who deteriorate with COVID-19 are deteriorating due to progression of their viral disease and this will not be able to be picked up by the pulse oximeter although most pulse oximeters do display a heart rate. Patient empowerment may be a key factor in the early identification of the critically ill patient with COVID-19.

## Competing interests

The author declares no competing interests.
